# Soy Protein Pressed Gels: Gelation Mechanism Affects the In Vitro Proteolysis and Bioaccessibility of Added Phenolic Acids

**DOI:** 10.3390/foods10010154

**Published:** 2021-01-13

**Authors:** Marina Marinea, Ashling Ellis, Matt Golding, Simon M. Loveday

**Affiliations:** 1 Riddet Institute, Massey University, Palmerston North 4442, New Zealand; A.Ellis@massey.ac.nz (A.E.); m.golding@massey.ac.nz (M.G.); simon.loveday@agresearch.co.nz (S.M.L.); 2 School of Food and Advanced Technology, Massey University, Palmerston North 4442, New Zealand; 3 Food and Bio-Based Products Group, AgResearch Limited, Palmerston North 4442, New Zealand

**Keywords:** soy protein gels, firm tofu, protocatechuic acid, coumaric acid, glucono δ-lactone, magnesium sulphate, bioaccessibility

## Abstract

In this study, a model system of firm tofu (pressed gel) was prepared to study how the coagulation mechanism—acidification with glucono δ-lactone (GDL) or coagulation with magnesium sulphate (MgSO_4_)—affected the physical properties of the gels along with their in vitro proteolysis (or extent of proteolysis). The two types of gels were also fortified with 3.5 mM protocatechuic (PCA) and coumaric acid (CMA) to test whether they can be used as bioactive delivery systems. Texture analysis showed that all MgSO_4_-induced gels (fortified and control) had a higher hydration capacity and a weaker texture than the GDL-induced gels (*p* < 0.05). MgSO_4_ gels had almost double proteolysis percentages throughout the in vitro digestion and showed a significantly higher amino acid bioaccessibility than the GDL gels (essential amino acid bioaccessibility of 56% versus 31%; *p* < 0.05). Lastly, both gel matrices showed a similar phenolic acid release profile, on a percentage basis (~80% for PCA and ~100% for CMA). However, GDL gels delivered significantly higher masses of bioactives under simulated intestinal conditions because they could retain more of the bioactives in the gel after pressing. It was concluded that the coagulation mechanism affects both the macro- and microstructure of the soy protein pressed gels and as a result their protein digestibility. Both pressed gel matrices are promising delivery systems for bioactive phenolic acids.

## 1. Introduction

Phenolic compounds are secondary metabolites of plants, and have various health benefits, such as antioxidant [1], anti-inflammatory [2], antimicrobial [3], anti-allergenic [4], anticancerogenic [5], and neuroprotective properties [6]. However, the bioavailability of the phenolics is low and it is not certain that they reach their target in the human body after consumption [7]. Over the last few years, research has shown that food structure and matrix can affect nutrient uptake and bioavailability [8,9].

Recently, there has been a systematic attempt to answer this question by studying the release (bioaccessibility) of native or added phenolics from various food matrices. Some of the existing food formats that have been fortified with phenolics are dairy products, such as milk [10,11], yoghurt [10,12,13,14,15,16], cheese [10,17,18,19,20], and ice cream [21]. The food structure can either facilitate the release of polyphenols or impede it. Some types of soy protein gels, have been proposed as good vehicles for modulating the release of riboflavin under in vitro digestion conditions [22,23] and it would worthwhile to investigate them further. The advantage of soy proteins is that they contain a balanced composition of polar, hydrophobic, and charged amino acids [24] that might allow the incorporation of a range of different bioactives.

Soy and its products are good sources of protein and provide adequate amounts of essential amino acids [25]. Soy products are a source of endogenous polyphenolics such as the isoflavones genistein and daidzein [26]. Tofu is the most popular and versatile product made from soymilk. Tofu is a nutrient-dense food, cholesterol-free, low in saturated fat, and packed with a good portion of minerals [27]. Tofu can be divided into two main categories, unpressed (silken) and pressed (firm) tofu, which differ in texture. The pressing process during the production of the firm or extra-firm tofu considerably alters the composition of the gel, i.e., protein and moisture content [28], and as a consequence, their physical characteristics. In addition, the type of coagulant used (acid or salt) during the production of tofu, affects the textural properties and the retention of isoflavones in firm tofu [29]. It is not clear, however, whether the type of coagulant or the physical characteristics of the soy protein gels can affect the extent of proteolysis and the bioaccessibility of added phenolics. Therefore, this was the main focus of this research.

Previous research that studied the effect of different coagulation mechanisms on the in vitro proteolysis of soy protein gels, focused on unpressed soy protein gels and did not offer insights on the microstructure of the gels [30]. Kozu et al. [31] demonstrated that the gel particles of soft tofu were disintegrated more easily and quickly than the firm tofu during in vitro processing. In a recent study, Reynaud et al. [32] found that soy proteins from a soya juice matrix were more prone to in vitro proteolysis than fresh tofu. Although the two soy matrices had a different protein concentration, the authors concluded that the food matrix had a strong influence on the protein digestibility [32]. Thus, the physical characteristics of the soy protein gels might affect their protein digestibility. The latter is of great importance for people consuming soy products as a main source of proteins.

To our knowledge, there are no available studies on protein digestibility of pressed soy protein gels or their use for studying phenolic acid bioaccessibility. In this study, we developed a firm tofu model system to investigate, firstly the impact of two different gelation mechanisms (acidification using glucono δ-lactone (GDL) and salt bridges using MgSO_4_) on the physical properties and microstructure of pressed soy protein gels as well as the effect on proteolysis patterns, and secondly, to explore how the two types of coagulation influence the bioaccessibility of added phenolic acids and thus, the potential of firm tofu as a carrier of biofunctional compounds for health improvement. Finally, the effect of added phenolic acids on the physical properties of gels was also studied.

## 2. Materials and Methods

Soy protein isolate (Wilpro G300) purchased from Wilmar International (Singapore). The coagulants; glucono-δ-lactone (GDL), magnesium sulphate (MgSO_4_ × 7H_2_O), the in vitro digestion materials; pepsin (P7000), pancreatin (P7545), and bile extract (B8631) that were of porcine origin, the pepsin inhibitor pepstatin A (P5318),the phenolic acids protocatechuic (37580, purity ≥ 97%) and o-coumaric acid (H22809, purity 97%), ortho-phtaldialdehyde (OPA P0657), L-serine (S4500), sodium dodecyl sulphate (SDS 436143), 1,4-dithiothreitol (DTT D0632), sodium tetraborate (BORAX), sodium azide and L-Norvaline were purchased from Sigma-Aldrich (Saint Louis, MO, USA). The trypsin inhibitor 4-(2-aminoethyl) benzenesulfonyl fluoride (AEBSF) hydrochloride (ab141403) was obtained from abcam (Melbourne, Australia). Ethanol, methanol, acetonitrile, and trifluoroacetic acid purchased from Merck (Auckland, New Zealand).

### 2.1. Preparation of Soy Protein Gels

#### 2.1.1. Soy Protein Isolate Composition

The protein content of the soy protein isolate (SPI) powder was determined using the Kjeldahl method [33] with a conversion factor of 6.25 and the moisture content determined by oven-drying at 105 °C for 24 h.

#### 2.1.2. Pressed Soy Protein Gels (Firm Tofu Model System)

Conditions for preparing pressed soy gels were chosen after preliminary optimization experiments (data not shown), on the basis that the gels formed with this method had high firmness. Approximately 850 g of 4.5% (*w*/*w*) SPI dispersion was heated in a water bath at 80 °C for 15 min (pre-treatment step) and subsequently cooled at room temperature until a final temperature of approximately 25 °C was reached. Then, approximately 10.3 g of o-coumaric (CMA) and protocatechuic acid (PCA) ethanolic solution (300 mmol/kg) or ethanol (in control gels) was added to the SPI dispersion, and the mixture was stirred for 5 min at room temperature. Both PCA and CMA were easily dissolved in pure ethanol after 2–5 min of vortexing at room temperature.

A mass of approximately 28 g of concentrated GDL or MgSO_4_ (950 mmol/kg) solution was poured into the mix and agitated gently (coagulant step) and subsequently heated at 80 °C for 30 min in a water bath (gelation step). The final concentration of the coagulants was approximately 30.32 ± 0.38 mmol/kg and of the phenolic acids 3.54 ± 0.07 mmol/kg.

The gels were then cooled down at room temperature for 30 min and poured into a plastic tofu press mold with dimensions 14 cm × 10 cm × 9 cm (Mangocore, Seattle, WA, USA). After the gels were transferred in the tofu mold, different weights were placed on top in a sequence of 700 g for 15 min, then 1500 g for 30 min, then 2300 g for 60 min. These weights were equivalent to pressures of 6.5, 13.8, and 21.3 g force/cm^2^ respectively, and the sequence of increasing weights allowed for better shaping of gels. After pressing, the whey was drained off, and parameters, such as final weight and whey pH were recorded. The gels were stored at 5 °C before further analysis.

### 2.2. Characterisation of Gels

#### 2.2.1. Composition

The protein content of the SPI and the gels was determined using the Kjeldahl method [23] with a conversion factor of 6.25 and the moisture content determined by oven-drying at 105 °C for 24 h. The pH of the whey serums was measured using a benchtop Orion 3-star, pH-meter (Thermo electron corporation) and the ζ-potential was measured using a Zetasizer (Nano ZS, Malvern Instruments Ltd., Malvern, United Kindom) at 25 °C using a DTS1060 cell. The yield of the gels was calculated from the following equation (Equation (1)).

(1) $$\mathrm{Yield}\;\left(\%\right)\;=\frac{\mathrm{Weight}\;\mathrm{of}\;\mathrm{gel}}{\mathrm{Weight}\;\mathrm{of}\;\mathrm{SPI}\;\mathrm{dispersion}}\;\times \;100$$

#### 2.2.2. Retention of Phenolic Acids in the Curd

The retention of the phenolic acids (PA), protocatechuic acid, and coumaric acid in the gels after pressing was calculated by subtracting the PA amount detected in whey (Equation (2)), from the total concentration of PA initially added to SPI dispersion, using the following equation (Equation (3)). The total amount of PA in the whey serum was calculated with the standard curves’ linear equations obtained by high-performance liquid chromatography (HPLC). All samples were diluted with 3 volumes of pure ethanol, then were centrifuged for 5 min at 11,000 rcf. The supernatant was immediately filtered with polytetrafluoroethylene (PTFE) syringe filters, 0.22 μm (NTSF2513-4, ThermoScientific, Auckland, New Zealand). All the samples were stored at −20 °C prior to further analysis. Each of these experiments was performed in triplicate.

(2) PA in the gel = Mass of added PA (mg)−Mass of PA found in whey serum (mg)

(3) $$\mathrm{Retention}\;\left(\%\right)\;=\;\frac{\mathrm{PA}\;\mathrm{in}\;\mathrm{gel}\;(\mathrm{mg})}{\mathrm{Total}\;\mathrm{amount}\;\mathrm{of}\;\mathrm{added}\;\mathrm{PA}\;(\mathrm{mg})}\;\times \;100$$

#### 2.2.3. HPLC Conditions

The recovery of the PA in whey was analyzed using with LC-20AD, Prominence UFLC, Shimadzu, Japan. Data analysis was performed with LabSolutions software (version 5.73, Shimadzu Corporation, Kyoto, Japan). Samples (5 μL) injected onto a Grace™ Alltech™ Prevail™ column, 150 mm × 4.6 mm i.d.; particle size 5.0 μm (Thermo Fisher Scientific, Auckland, New Zealand). The column oven temperature was set at 25 °C. Elution of phenolic acids (0.5 mL/min) was performed using aqueous trifluoroacetic acid (TFA) solution (0.02% *v*/*v*) (eluent A) and methanol containing 0.02% (*v*/*v*) TFA (eluent B) [34]. The elution gradient was as follows: 0–5 min, 25% B; 5–10 min, 25–30% B; 10–16 min, 30–45% B; 16–18 min, 45% B; 18–25 min, 45–80% B; 25–30 min, 80% B; 30–40 min, 80–25% B; 40–50 min, 25% B. Protocatechuic acid was recorded at 295 nm and coumaric acid at 325 nm [34]. Quantification of phenolic acids in the gels was based on the calibration curves that were obtained by the standard compounds (purity ≥ 96%) in a concentration range between 0.01 to 0.13 mg/g. For the purpose of the calibration curve, phenolic acids were diluted in an aqueous solution of 63% (*w*/*w*) ethanol.

#### 2.2.4. Texture Profile Analysis

Pressed gels were removed from the refrigerator and cut with a stainless cylinder cutter with a diameter of 22 mm and a height of 1.5 cm. Fracture stress and strain were measured with a TA.XT plus (Stable Micro Systems, Surrey, UK) mounted with a 51 mm flat cell loaded with 50 kg. Samples were compressed to 80% of their initial height at a constant deformation speed of 4 mm/s. The experiment was performed in triplicate on each of the 6 gels from different batches. The mean values of true stress (σ) (Equation (4)) and Hencky’s strain (ε_h_) (Equation (5)) were calculated according to Steffe [35]:

(4) $$\sigma \;=\frac{F}{\mathrm{Ao}}\;\left(\frac{L}{\mathrm{Lo}}\right)\;\left(\mathrm{Pa}\right)$$

(5) $$\varepsilon_{h}\;=\;\int_{\mathrm{Lo}}^{L}\frac{\mathrm{dL}}{L}=\ln\frac{L}{\mathrm{Lo}}\;\left(-\right)\;$$

F (N) is the force recorded per unit of sample area $\mathrm{Ao}\;\left(m^{2}\right)$. The stress is corrected by including the ratio of the cylinder (sample) lengths in the stress calculation. Lo is the initial length and L the deformed length of the sample. The true strain is negative for compression experiments but is expressed as an absolute value. The fracture stress was measured by the local maximum of the stress over the strain curve [36]. Fracture strain is the one that corresponds to the fracture stress [36]. Young’s modulus, E (Pa) was calculated from the linear part of the stress over strain curve within the region of 0.05–0.01 of fracture strain and is defined according to the Equation (6):

(6) $$E\;=\left(\frac{d\sigma }{{d\varepsilon }_{h}}\right)$$

#### 2.2.5. Scanning Electron Microscopy (SEM)

Samples were cut into small pieces (2–3 mm) and then were fixed in 0.1 M phosphate buffer containing 3% (*w*/*v*) glutaraldehyde and 2% (*w*/*v*) formaldehyde (pH 7.2) for 24 h at room temperature. The samples were washed three times for 10 min each in 0.1 M phosphate buffer (pH 7.2) followed by ethanol dehydration using a series of solutions of increasing ethanol concentrations: once at 25, 50, 75, and 95% for 10 min each time and at 100% for 1 h. All the samples were critical point dried in a Polaron E3000 series II apparatus, using liquid carbon dioxide as the critical point fluid and 100% ethanol as the intermediary. Samples were torn to expose the inside of the cube and mounted on the aluminum stubs using double-sided tape. The dried samples were then sputter-coated with 100 nm of gold (Bal-Tec SVD050, Los Angeles, CA, U.S.A. and viewed in the scanning electron microscope (Quanta 200 Environmental) from FEI Co. (Hillsboro, OR, USA) at an accelerating voltage of 20 kV.

#### 2.2.6. Transmission Electron Microscopy (TEM)

Tubes of samples (2–3 mm) were fixed in 0.25 M glutaraldehyde in a 0.1 M sodium cacodylate buffer at pH 7.2 for 16 h at 4 °C. After fixation, specimens were rinsed 3 times in 0.1 M sodium cacodylate buffer, before post-fixation with 1% osmium tetroxide in 0.1 M sodium cacodylate buffer for 1 h at room temperature. After post-fixation, the samples were rinsed 3 times in 0.1 M sodium cacodylate buffer. The samples were dehydrated through a graded series of acetone 45 min in each step (25%, 50%, 76%, 95%, 100%) and were embedded in 50:50 resin:acetone and incubated overnight. The mixture was replaced with 100% epoxy resin (Procure 812, ProSciTech, Thuringowa Central, Australia) and incubated for 8 h (repeated twice). The samples were then embedded in molds with fresh resin and cured at 60 °C for 48 h.

Light microscope sections of 70 nm thickness were cut from the resin blocks and were mounted on copper grids. Grids were stained in saturated uranyl acetate in 50% ethanol for 4 min, washed with 50% ethanol and MilliQ water, and then stained in lead citrate [37] for a further four minutes and washed with MilliQ water. Samples were viewed using an FEI Tecnai G2 Spirit BioTWIN transmission electron microscope (FEI Corp., Brno-Černovice, Czech Republic). Twenty TEM images were taken at a magnification of ×20,500 on different parts of each gel. Image processing and analysis were conducted using ImageJ (1.52f, National Institute of Health, Bethesda, MD, USA) The porosity of the protein network of the gels was characterized according to the procedure proposed by Silva et al. [38]. Briefly, a black top-hat filter (200 × 200) that can be found from MorphoLibJ plugin was used first and then the Otsu automated threshold was applied, which resulted in a binary image, which allowed the measuring of the total area of the black and white area.

### 2.3. In Vitro Digestion Static Protocol

In vitro digestion experiments were conducted as stated in the INFOGEST protocol with minor modification [39].

#### 2.3.1. Oral Phase

The gel bolus was obtained by mixing 5 g of shredded gelled samples with 4 mL of simulated salivary fluid (SSF), 0.025 mL of 0.3 M CaCl_2_ and 0.975 mL of distilled water in an amber glass bottle for 5 min. The salivary a-amylase was excluded from our experiments due to a lack of starch in the samples [40]. The gel bolus was then kept at 37 °C under shaking at 40 rpm in a shaking water bath. A volume of 0.5 mL of digesta was withdrawn at the end of the oral phase.

#### 2.3.2. Gastric Phase

The 9.50 g of the simulated oral bolus were mixed with 7.60 mL of simulated gastric fluid (SGF), 4.8 μL of 0.3 M CaCl_2_, and the pH was adjusted to 3.0 by adding 0.25 to 0.55 mL of 1 M HCl. The rest of the volume was filled with 0.69 to 0.39 mL of distilled water and 0.95 mL of porcine pepsin stock solution (40,000 U/mL) to achieve an activity of 2000 U/mL in the final mixture. Then, the mixture was incubated for 2 h in a shaking water bath at 37 °C (40 rpm). A volume of 0.4 mL was withdrawn at times 30, 60, and 120 min of the gastric processing for further analysis.

#### 2.3.3. Intestinal Phase

The 17.80 g of the simulated gastric chyme was mixed with 9.76 mL of simulated intestinal fluid (SIF), 2.24 mL of bile (approximately 160 mM in SIF), and 36 μL of 0.3 M CaCl_2_, the pH was adjusted to 7.0 by adding 0.30 to 0.50 mL of 1 M NaOH. The rest of the volume was filled with 0.80 to 1.00 mL of distilled water and 4.47 mL of pancreatin stock solution (800 U/mL) to achieve a trypsin activity of 100 U/mL in the final mixture. Both pancreatin and bile salts are not readily dissolved in simulated fluids, thus they were vortexed and magnetically stirred for approximately 20 min under cool conditions, prior to intestinal processing. The simulated digesta was incubated for 2 h in a shaking water bath at 37 °C (40 rpm). A volume of 0.4 mL was withdrawn at different time intervals 5, 15, 30, 60, and 120 min from the intestinal phase for further analysis.

#### 2.3.4. Sample Collection

In case of the control trials (gels without phenolic acids), the enzymatic reactions were stopped using pepstatin A at a final concentration of 0.5 mg/mL for the gastric samples and ABSF of 0.1 M for the intestinal samples, respectively [40]. After the addition of the inhibitors, the samples the immediately vortexed and centrifuged for 5 min at 11,000 rcf.

Gels containing phenolic acids were treated without inhibitors due to adverse interaction. In particular, it was observed that the addition of ABSF reduced the levels of protocatechuic acid in the intestinal phase.

The digesta of the fortified gels were immediately centrifuged for 5 min at 11,000 rcf, then the supernatant was transferred to a pre-weighed tube containing two volumes of ethanol, the tube was weighed again to determine the exact amount of sample and finally, it was centrifuged again for 3 min at 11,000 rcf. The dissolved samples were immediately filtered with 0.22 μm syringe filters (PTFE). All the samples were stored at −20 °C prior to further analysis. Each of these experiments was performed in triplicate.

### 2.4. Digesta Analysis

#### 2.4.1. Degree of Protein Hydrolysis

The degree of protein hydrolysis was determined using the o-phthaldialdehyde (OPA) assay as reported by Nielsen and co-workers [41]. The OPA solution consisted of 80 mL of 125 mM sodium tetraborate (BORAX) and 4.3 mM of sodium dodecyl sulphate (SDS), 2 mL of 300 mM OPA in methanol, and 2 mL of 285 mM dithiothreitol (DTT). The final volume was adjusted to 100 mL with distilled water. The assay was performed by mixing 0.1 mL of diluted digesta with 1 mL of OPA solution. The mixture was vortexed and kept at room temperature for two minutes, followed by measurement of absorbance at 340 nm. The concentration range that was used for the calibration curve of L-serine was 0.007–0.144 mM. The degree of protein hydrolysis (DH%) was calculated using Equation (7) [42]:

(7) $$\mathrm{DH}\;(\%)\;=\;\frac{{\mathrm{NH}}_{2}\left(\mathrm{final}\right){-\;\mathrm{NH}}_{2}\left(\mathrm{initial}\right)}{{\mathrm{NH}}_{2}\left(\mathrm{acid}\right){-\;\mathrm{NH}}_{2}\left(\mathrm{initial}\right)}\;\times \;100$$

where NH_2_ (final) is the concentration of free amino groups of the digested samples, NH_2_ (initial) is the concentration of free amino groups in the undigested sample (oral phase), and NH_2_ (acid) is the total amount of free amino groups in the gel samples after acid hydrolysis as it is described in Section 2.4.3. The total free amino acid concentration was found on average 7.05 ± 1.065 mmol serine equivalents/g of protein for GDL-induced gels and 7.71 ± 0.713 mmol serine equivalents/g of protein for MgSO_4_-induced gels. The experimental values were comparable with the theoretical value (7.67 mmol of total amino acids/g of protein) as it was calculated from the amino acid composition of soybeans reported in the literature [43].

#### 2.4.2. Free Amino Acid Determination

Free amino acid (FAA) determination was based on the protocol from Liu and co-authors [44]. Briefly, the samples that were collected after in vitro digestion were deproteinized by ultrafiltration (Vivaspin 3000 MWCO, Sartorius, Göttingen, Germany) at 14,000 rcf, 20 °C for 1 h. Norvaline was used as an internal standard. Amino acid concentrations were determined with the use of HPLC (Agilent 1200SL, Agilent Technologies, Santa Clara, CA, USA), Eclipse plus C18 Narrow Bore column, 150 mm × 2.1 mm i.d.; particle size 3.5 μm (Agilent Technologies, Santa Clara, CA, USA). The column oven temperature was set at 40 °C. Elution of amino acids (0.42 mL/min) was performed using an aqueous solution consisting of 10 mM sodium phosphate dibasic, 10 mM Borax, and 0.5 mM of sodium azide in pH 8.2 (eluent A) and a mixture of methanol/acetonitrile/water in ratio 45/45/10 (eluent B). The elution gradient was as follows: 0–3 min, 100% A; 3 min, 100% A; 3–10.4 min, 100–81.5% A; 10.4–23.0 min, 81.5–43.0% A; 23.0–23.1 min, 43.0–0% A, 23.1–28.0 min, 0% A, 28.0–28.5 min, 0–100% A, 28.5–30 min, 100% A.

#### 2.4.3. Total Content of Free Amino Acids

Gel samples (undigested) were hydrolyzed using hydrochloric acid (6 mol/L) containing 0.1% (*w*/*v*) phenol at 115 °C for 24 h under vacuum. Tryptophan could not be detected following acid hydrolysis due to oxidative degradation.

#### 2.4.4. Phenolic Acids Release

The quantification of phenolic acids (PA) in digesta was obtained by HPLC analysis. The PA release was calculated according to Equation (8):

(8) $$\mathrm{PA}\;\mathrm{release}\;(\%)\;=\;\frac{\mathrm{Mass}\;\mathrm{of}\;\mathrm{PA}\;\mathrm{detected}\;\mathrm{in}\;\mathrm{digesta}\;(\mathrm{mg})}{\mathrm{Total}\;\mathrm{mass}\;\mathrm{of}\;\mathrm{PA}\;\mathrm{retained}\;\mathrm{in}\;\mathrm{curd}\;\mathrm{after}\;\mathrm{pressing}\;(\mathrm{mg})}\;\times \;100$$

### 2.5. Statistical Analysis

All experiments were conducted in triplicate, the results were reported as means ± standard deviations of the measurements. Data were analyzed using Minitab 17 statistical software (Minitab Inc., State College, PA, USA). The statistical analysis was conducted using the Student *t*-test for two-sample comparison and the linear regression model (ANOVA) for multi-factor comparison followed by Tukey’s pairwise test. The correlations were considered significant when *p* < 0.05.

## 3. Results and Discussion

### 3.1. Physical Properties of Control and Fortified Gels

#### 3.1.1. Gel Characteristics

As can be seen from Table 1, the gelation mechanism affected some of the physical characteristics of the gels. Water and whey were expelled during the pressing of the curd, which reduced the moisture content. Whey proteins mainly consist of soluble proteins that do not solidify during the curding process [28]. Gels produced by GDL had a lower moisture content than the ones produced by MgSO_4_ (*p* > 0.05) and thus, lower yield. In other words, there was an inverse relationship between the yield and the moisture content of the curd (Table 1).

MgSO_4_-induced gels had a significantly lower protein concentration than the GDL gels (*p* < 0.05), which is a consequence of the higher water retention capacity of these gels. Moreover, the soluble fraction of proteins that drained off with the whey was significantly higher (*p* < 0.05) than the GDL-induced gels. The protein content and participation in the network can largely affect the stiffness of the final curd [45].

Even though there were large differences in whey pH (*p* < 0.05) between the GDL and MgSO_4_ gels, the net charge of all the wheys was slightly negative (Table 1). The whey pH of the GDL-induced gels was approximately 4.7, which is close to the isoelectric point of the soy proteins, while in MgSO_4_-induced gels the pH was between 6.1 and 6.4. At high ionic strength, the charge is screened, the electrostatic repulsion is reduced and as a result the protein solubility is reduced too. Other studies have indicated that the solubility of soy proteins strongly decreased at CaCl_2_ concentrations ≤ 0.1 M [46,47], which is within the range of MgSO_4_ concentration used in this work.

Phenolic acid addition (PCA and CMA) did not alter the characteristics of the gels to a large extent. Han, Britten, St-Gelais, Champagne, Fustier, Salmieri, and Lacroix [18] found a significant reduction in the hydration capacity of cheese curd after the addition of various polyphenols (PP). This phenomenon was explained by the potential hydrophobic interaction of phenolics with milk proteins which, thereby, can disrupt the amino acid side chain interactions and reduce the quantity of the entrapped water [18]. Nevertheless, the only significant effect observed in our system was the reduction of the whey pH in MgSO_4_-induced gels, by approximately 0.3 units (*p* < 0.05) (Table 1). This pH change was expected given phenolic acids are weak acids [48].

The retention of the phenolic acids is the parameter that determines the final concentration of the bioactive in the curd. According to Table 1, the retention of CMA was higher in both types of gels, whereas in the case of GDL-induced gels, CMA retention was significantly higher than PCA (58.8% versus 40.2%; *p* < 0.05). The difference in phenolic acids retention can be attributed to their polarity. Helal, Tagliazucchi, Verzelloni, and Conte [19] found a positive correlation between added polyphenols (PP) retention in cheese curd and binding affinity with caseins and a negative correlation with the PP hydrophilicity [19]. Similarly, the native aglycon isoflavones in soybean were more likely to precipitate during the soy protein curding process than the glucoside derivatives [49]. Both studies indicate that the more hydrophilic the phenolics, the more likely to be washed out with whey and water during pressing, which agrees with our results (Table 1). PCA was the most hydrophilic and CMA the most hydrophobic phenolic acid with LogP values of ~0.86 and ~1.90, respectively [50,51].

#### 3.1.2. Textural Properties

Texture analysis was performed by measuring fracture stress (kPa), Young’s modulus (kPa), and fracture strain (-) of the gels. According to previous studies, fracture properties are important for understanding the mastication process as well as influencing disintegration during digestion [52].

**Figure 1:**
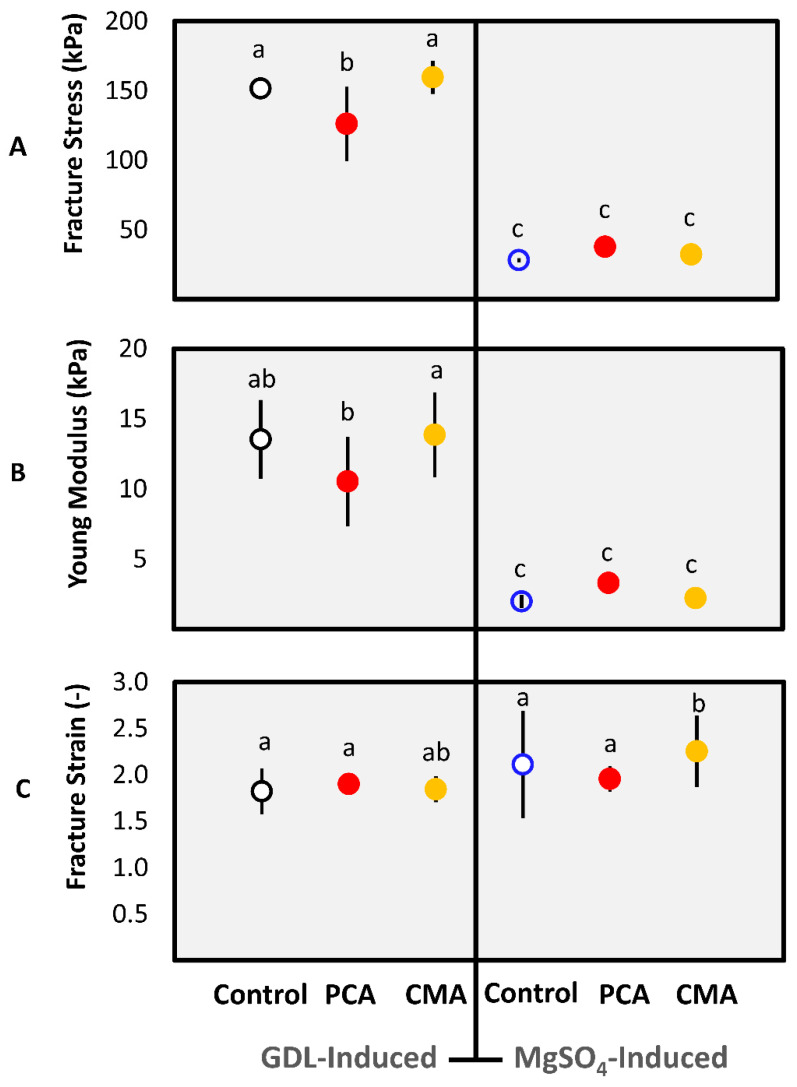
Textural properties of pressed soy protein gels induced by GDL and MgSO4 with added phenolic acids: (**A**) Fracture stress (kPa), (**B**) Young’s modulus (kPa), and (**C**) fracture strain, at 80% of deformation. The red color bullets indicate the addition of the PCA and the yellow the addition of CMA. Values were represented as means ± standard deviations (*n* ≥ 8). Different letters indicate significant differences (*p* ≤ 0.05).

The results showed that the gelation mechanism affects fracture stress and Young’s modulus significantly (*p* < 0.05) (Figure 1A,B). On the contrary, all the gel samples regardless of the gelation mechanism or the addition of phenolic bioactives were similar in fracture strain. The only exception was the CMA-MgSO_4_ gel that gave a slightly higher fracture strain (*p* < 0.05) (Figure 1C). The last parameter is a measure of gel’s elongation at break. The higher the fracture strain value, the more extensible is the gel. Therefore, the brittleness of the different gels is also similar (1⁄εfr) [53].

Fracture stress is a measure of the strength of a material [53] and is an indication of the force required to deform the gel permanently upon compression [36]. Furthermore, Young’s modulus is a measure of the stiffness of the gels without damaging the structure [53]. GDL-induced gels were significantly (*p* < 0.05) stiffer or less elastic than the MgSO_4_-induced gels. An explanation of this phenomenon is that the protein and water retained in GDL-induced gels were higher than in the case of the MgSO_4_ gels (Table 1). However, this large difference in the textural characteristics is more likely due to interactions at a molecular level.

Visually, the MgSO_4_-induced gels were smooth with a spreadable texture, whereas GDL-induced gels were coarse and brittle. These results can be explained by the significantly lower moisture content of the GDL gels compared to the MgSO_4_ gels (*p* < 0.05). Urbonaite et al. [54] showed that the higher the stiffness of MgSO_4_/MgCl_2_ soy protein gels, the lower the water holding capacity of the gel network. The divalent cations (Ca^2+^, Mg^2+^) of the salts bind or cross-link to the proteins’ negatively charged groups (-COO^-^) forming bridges, which entraps water within the gel. The dominant forces in this mechanism are ionic binding due to the salt addition and hydrophobic interactions. In the case of the GDL, the release of the acidifier (gluconic acid or H^+^) gradually neutralizes the negative charges of the proteins resulting in the formation of water layer [55] around polypeptides and not within the gel structure.

The effect of different coagulants on the textural properties of soy protein gels has been observed in many studies [29,30,56,57,58]. Rui, Fu, Zhang, Li, Zare, Chen, Jiang, and Dong [30] found that soy protein gels with different GDL concentration were significantly harder than MgCl_2_ and microbial transglutaminase induced gels. Lu et al. [59] compared the ability of various coagulants to form firm tofu and they found that the GDL-induced tofu was the firmest and the rubberiest. Moreover, Prabhakaran, Perera, and Valiyaveettil [29] found that firm tofu produced by MgSO_4_ was significantly weaker than tofu produced by other salts. Similarly, Li Tay, Yao Tan, and Perera [56] found that that among four salt coagulants the weakest soy protein gels were formed by MgSO_4_ and the strongest by CaCl_2_. In contrast Deman [58], found that the soy protein gels obtained by MgCl_2_ were harder than GDL and MgSO_4_. These differences could be due to the coagulant concentrations used in different studies or more specifically due to soy protein to coagulant ratio differences.

Finally, the addition of PCA and CMA affected the textural properties of both types of gels. PCA reduced the strength and stiffness of the GDL-induced gels but had the opposite trend in MgSO_4_-induced gels. CMA affected only the MgSO_4_ gels in both parameters (Figure 1A,B). In addition, the pH of the gels can affect the phenolic acid properties, since the pk_a_ of the -COOH group of PCA and CMA is between 4.0 and 4.3 [50,51]. GDL-induced gels had a low pH close to the pk_a_ of the phenolic acids, which were in their protonated form. On the other hand, the pH of MgSO_4_-induced gels was above 6, which means that the phenolic acids were negatively charged. The state of the phenolic acids in the different gels is important because it can determine their reactivity, which might be associated with the effect on the textural properties found in Figure 1.

#### 3.1.3. Microstructural Characteristics of Gels

**Figure 2:**
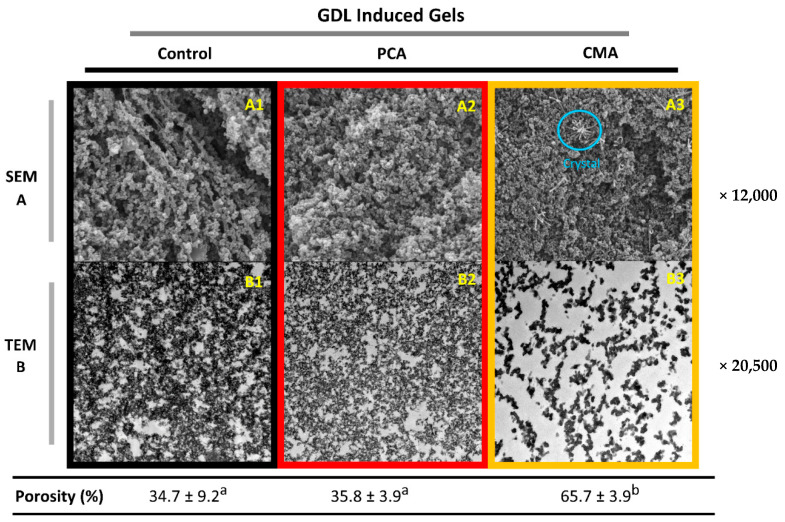
Microstructural characteristics of soy protein pressed gels induced by GDL. The SEM micrographs were presented on the (**A**) row and the TEM micrographs on the (**B**) row. GDL-induced gels without the addition of bioactives (**A1**,**B1**), gels with added PCA (**A2**,**B2**) and CMA (**A3**,**B3**). The values of porosity were represented as means ± standard deviations (*n* = 20). Different letters indicate significant differences (*p* ≤ 0.05).

**Figure 3:**
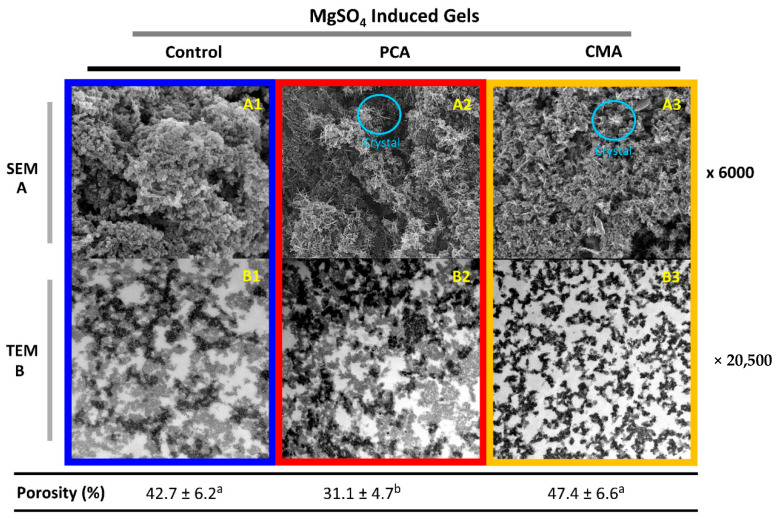
Microstructural characteristics of soy protein pressed gels induced by MgSO_4_. The SEM micrographs were presented on the (**A**) row and the TEM micrographs on the (**B**) row. Gels without the addition of bioactives (**A1**,**B1**), gels with added PCA (**A2**,**B2**) and CMA (**A3**,**B3**). The values of porosity were represented as means ± standard deviations (*n* = 20). Different letters indicate significant differences (*p* ≤ 0.05).

In Figure 2 and Figure 3, the SEM and TEM micrographs of the gels can be found. The three-dimensional protein network of the gels’ surface can be seen with the SEM imaging in Figure 2A and Figure 3A, while the inner structure of the gels is more obvious with TEM imaging Figure 2B and Figure 3B. The dark areas of the TEM images correspond to the protein network or aggregates and the white areas to the aqueous phase.

Overall, particulate networks can be seen in all cases, formed through a rapid aggregation which occurred in both gelation mechanisms. However, the protein networks between GDL- and MgSO_4_-induced gels appear very different. Although the gels’ microstructure consisted of a coarse network of spherical particles in both types of gels, the diameter of the particles and the density of the network varied. On the one hand, GDL-induced gels (both control and PCA) (Figure 2(B1,B2)) were dense with a more “curly” protein network and with many intermediate tiny pores. In contrast, MgSO_4_-induced gels had larger, thicker aggregates surrounded by large pores (Figure 3(B1,B2)). However, the control GDL gel showed a more porous network on the surface (Figure 2(A1)) than the MgSO_4_ control gel, which was more compact and denser (Figure 3(A1)). Nevertheless, SEM is not the appropriate method of assessing the porosity since it only gives a view of the samples’ surface [38]. A range of TEM images was used for quantifying the porosity, based on the procedure described in Section 2.2.6.

The addition of PCA and CMA induced changes in both types of gels. Firstly, the addition of PCA increased the density of the MgSO_4_ gels network and reduced the porosity, significantly (Figure 3(B2)). This could explain the increase that was observed in yield stress and Young’s modulus (Figure 1A,B). It was found that the trend was the opposite in the GDL gels, following the addition of the PCA. The structure became less dense, but there was no significant change in the porosity (Figure 2(B2)). Similarly, this could explain the reduction in the yield stress and Young’s modulus that was observed for this gel.

We hypothesize that the addition of PCA interfered with the protein-protein interactions sites in in GDL-induced gels which might lower the density of the protein network and consequently the firmness of the gels. However, the effect of PCA was not significant enough in order to reduce the hydration capacity of the gels (Table 1). The addition of CMA induced more dramatic changes to the microstructure of both GDL and MgSO_4_ gels. Noticeably, the shape and size of the protein aggregates in gels with added CMA was identical, in both types of gels (Figure 2(B3) and Figure 3(B3)). Although the aggregates formed after the addition of CMA were more linear and thinner, which suggest less aggregation, there were no other effects on the gel characteristics.

Finally, crystal formation was observed on the surface of the MgSO_4_-PCA and MgSO_4_-CMA gels (Figure 3(A2,A3)) and some small evidence on the GDL-CMA gel (Figure 2(A3)). However, the crystal existence was not confirmed with X-ray diffraction experiments (results not shown), which might be an indication of an artefact during the SEM sample preparation.

### 3.2. Digesta Characterisation

#### 3.2.1. Degree of Proteolysis

**Figure 4:**
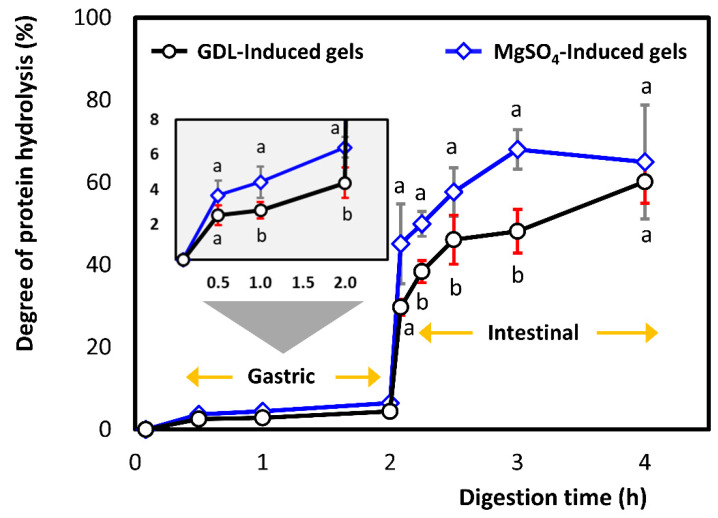
Degree of protein hydrolysis (%) of pressed gels (control) induced by GDL (○) and MgSO_4_ (◊) during in vitro simulation of digestion. The values were represented as means ± standard deviations (*n* ≥ 3). Different letters indicate significant differences (*p* ≤ 0.05).

Free amino acids and oligopeptides released during in vitro gastrointestinal digestion were measured using the OPA assay (Figure 4). The OPA reagent allows the estimation of the number of primary amino groups released during protein hydrolysis [60], which correlates with the proportion of amide bonds broken.

In the case of the GDL-induced gel, the amount of free amino acids and peptides liberated by the end of gastric processing was 0.32 ± 0.15 mmol serine equivalent/g of protein corresponding to 4.4 ± 0.9% of the total protein hydrolysis. Whereas, MgSO_4_-induced gels had a degree of proteolysis value of 6.4 ± 0.6% (*p* < 0.05). Pepsin is an endopeptidase that cleaves the proteins internally to smaller polypeptides. It has a higher specificity for hydrophobic/aromatic amino acids, and it typically digests 10–15% of dietary proteins in the stomach [61]. Our degree of hydrolysis values at the end of the gastric phase are comparable with the ones reported for fresh tofu using the INFOGEST protocol (DH ~2 to 4% after 120 min) [32]. The authors used the ninhydrin assay to measure the release of α-amino groups, which could explain the small differences compared to our results [32].

The addition of the pancreatin juice increased the extent of protein hydrolysis rapidly (Figure 4). Pancreatic juice contains a mixture of peptidases; both endo- and exo-peptidase, with various specificities, that results in the production of smaller peptides and free amino acids. After 5 min of intestinal processing, a large fraction of amide bonds had been broken in both samples corresponding to proteolysis percentages of 29.7 ± 1.9% for GDL and 45.1 ± 9.6% for MgSO_4_-induced gels (*p* < 0.05), respectively. After 2 h at intestinal conditions, 60.2 ± 5.2% and 64.9 ± 13.8% of protein from GDL and MgSO_4_ gels (*p* > 0.05) had been degraded into oligopeptides and/or free amino acids. Therefore, a steady increase in the release of the α-amino groups was observed in both types of gels, with MgSO_4_ gels having higher percentages throughout the in vitro processing.

Our final degree of proteolysis values (end of intestinal phase) were different than other articles studied soy protein gels matrices. Rui, Fu, Zhang, Li, Zare, Chen, Jiang, and Dong [30] reported DH between 80 to 90% in soy protein unpressed gels, while Reynaud, Lopez, Riaublanc, Souchon, and Dupont [32] reported DH of around 30% in fresh tofu (pressed gel). In general, there is some inconsistency in the degree of proteolysis values reported. Lamothe, Azimy, Bazinet, Couillard, and Britten [10] compared the protein digestibility of milk, yoghurt, and cheese. Although they found that cheese (pressed product) was more resistant to proteolysis [10], the reported values of protein digestibility were very high (~90%). In contrast, other studies on dairy products have reported a degree of hydrolysis below 40% for pressed cheese, such as mozzarella [40].

Some of the reasons for this variation are the following: firstly, compositional differences, especially protein content, which can affect the enzyme-to-substrate ratio (E:S) during in vitro digestion; secondly, the in vitro digestion protocol used; in many protocols, the enzyme activities were not estimated; and finally, the extent of enzyme autolytic activity, which is usually ignored.

Our blank in vitro digestion trials showed that the pancreatic extract used in this study was susceptible to autolysis. The protease autolytic activity reduces the proteolytic activity of the enzymes [62]. In addition, enzyme autolysis releases peptides and amino acids bearing alpha-amino groups, leading to inflation of OPA and amino acid bioaccessibility results. Qiao and co-workers studied the autolytic reaction of pepsin and pancreatic enzymes and they reported important findings [63,64]. We believe that the autolytic activity of the digestive enzymes is a very serious issue that needs to be researched further.

#### 3.2.2. Amino Acids Bioaccessibility

The amount of free amino acids (FAA) released at the end of the gastric and intestinal processing was measured with OPA pre-column derivatization followed by HPLC analysis (Section 2.4.2).

**Figure 5:**
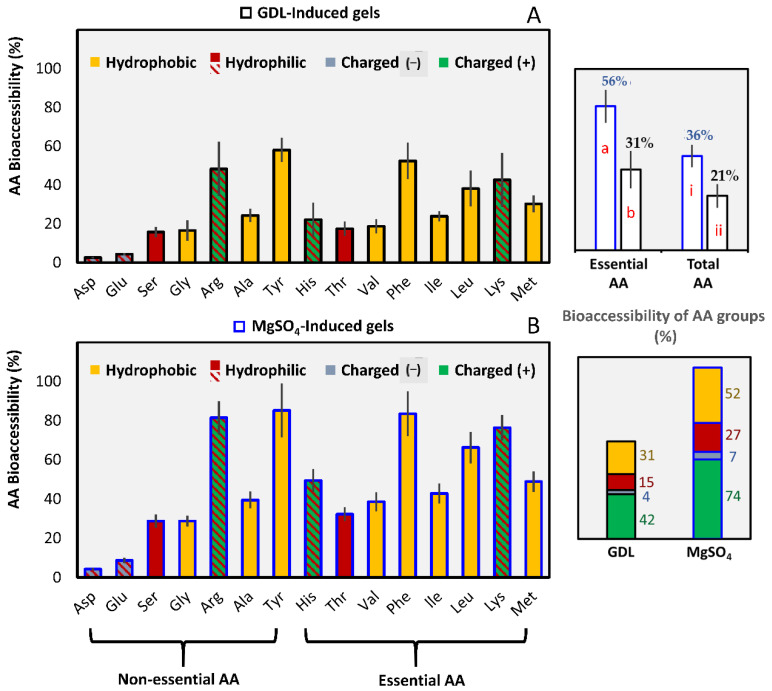
Bioaccessibility (%) of total, individual, and grouped amino acids at the end of the intestinal processing of the control pressed gels. The black frame around the bars correspond to GDL-induced gels (**A**) and the blue to MgSO_4_-induced gels (**B**). Amino acids were grouped according to Petsko and Ringe [65] and Damodaran et al. [66]. The values were represented as means ± standard deviations (*n* ≥ 3). Different letters (a, b) indicate a significant difference among essential AA and different Roman numerals indicate a significant difference between total AA (*p* ≤ 0.05).

The total amount of FAA released at the end of the gastric phase was negligible (0.71 ± 0.13% for GDL and 0.81 ± 0.05% for MgSO_4_ gels). The overall trend is similar to the degree of proteolysis results (Figure 5).

The release of all amino acids, both total and essential, in the end of the intestinal phase was considerably higher (*p* < 0.05) for the MgSO_4_ than the GDL-coagulated gels (total 36.4 ± 4.3%; 21.0 ± 4.6% and essential AA 55.6 ± 6.4%; 31.2 ± 7.3%, respectively) (Figure 5). Rui, Fu, Zhang, Li, Zare, Chen, Jiang, and Dong [30] found a similar trend for the salt-induced (MgCl_2_) soy protein unpressed gels, although the reported values were only slightly different than the GDL-induced gels.

All amino acids were less than 60% bioaccessible for GDL-induced gels, which was significantly lower than the MgSO_4_ gels (*p* < 0.05). However, the amino acid analysis profile showed a similar trend for both types of gels. Tyrosine, phenylalanine, and arginine were the most bioaccessible AAs with percentages between 85 to 81% for the MgSO_4_-induced gels and 58 to 48% for the GDL-induced gels (Figure 5). The same profile has been reported in soy protein gels before [30].

In addition, lysine, leucine, histidine, methionine, and isoleucine were moderately bioaccessible with percentages ranging between 76 to 43% for the MgSO_4_ gels and 22 to 43% for the GDL gels. The negatively-charged aspartic and glutamic acids were the least bioaccessible with percentages below 10% in both types of gels (Figure 5). Therefore, the small peptides that the aforementioned amino acids participate in cannot be further cleaved under the current in vitro digestion conditions [67]. Another hypothesis of the low Glu/Asp levels is that they might bind to larger materials because their carboxyl group is ionized under the intestinal conditions.

The release of the basic amino acids arginine and lysine was favored because they are the target of the specific action of both trypsin and carboxypeptidase B [61]. Similarly, tyrosine, phenylalanine, and leucine are the cleavage points of both pepsin and chymotrypsin [61].

Our results suggest that the gelation mechanism can significantly affect the protein digestion rates, which signifies the importance of gel’s physical properties. MgSO_4_-induced gels had a significantly softer texture, larger porosity, and a less convoluted protein network, which could increase the accessibility to the digestive enzymes and therefore render them more prone to proteolysis.

Our results suggest that the gelation mechanism can significantly affect the protein digestion rates, which signifies the importance of gel’s physical properties. MgSO_4_-induced gels had a significantly softer texture, larger porosity, and a less convoluted protein network, which could increase the accessibility to the digestive enzymes and therefore render them more prone to proteolysis.

Finally, a high concentration of free amino acids was found in our blank digestion trials. The concentration of some amino acids was higher in the blank digestions than in the gel samples (Table S1). Our results confirmed the autolytic reaction of the pancreatic enzymes, mentioned in Section 3.2.1, and showed that the extent of the autolytic reaction depends on the presence or absence of substrate and indicates that further research is needed.

#### 3.2.3. In Vitro Bioaccessibility of Phenolic Acids

**Figure 6:**
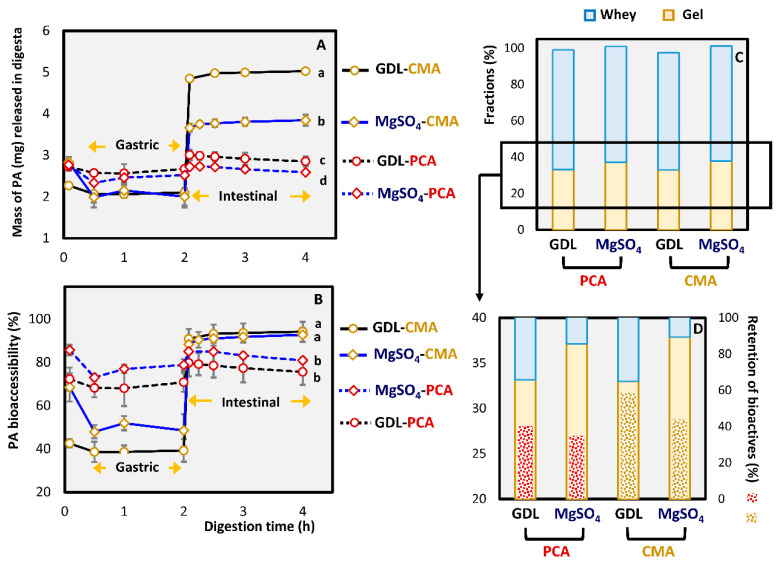
Release of added phenolic acids (PA); CMA and PCA from GDL- and MgSO_4_-induced pressed gels during in vitro digestion. Mass of bioactives released (mg) (**A**) and release percentage normalized with the mass of bioactives that remained in the gel after pressing (**B**). Fraction (%) of gel and whey serum after pressing (**C**) and magnification of image C with the differences in the retention (%) of phenolic acids in the gel after pressing (**D**). The values were represented as means ± standard deviations (*n* ≥ 3). Different letters indicate significant differences (*p* ≤ 0.05).

The release of phenolic acids during in vitro digestion was illustrated in two ways; the mass of bioactives detected in the liquid fraction of digesta (A) and the release percentage, based on the mass remaining within the gel after pressing (B). Although the release profiles of PCA and CMA from both MgSO_4_ and GDL-induced gels were not significantly different on a percentage basis (*p* > 0.05) (Figure 6B), GDL gels were superior (*p* < 0.05) (Figure 6A), because they retained more of the bioactives in the gel (Figure S1) and therefore released a greater mass of CMA and PCA in the intestinal phase. This statement is very important because it shows that the structure and texture of the gels do not significantly affect the relative (normalized) release rate of the two phenolic acids (*p* > 0.05). The trend observed in Figure 6A is a result of physical phenomena involved during gelation as a result of the coagulant mechanism (indirect effect). In summary, GDL coagulation produced a low yield of gel with high retention of phenolic acids, whereas MgSO_4_ gave higher gel yields but lower retention of phenolic acids (Figure 6C,D and Figure S1).

The most significant difference between the gels induced by different coagulants is that the MgSO_4_-induced gels tend to result in a higher bioactive release at the end of the oral phase (Figure 6A,B). This could be due to differences in the acidity and the protein content of the gel matrices that affects their buffering capacity. MgSO_4_ gels have significantly higher pH than the GDL gels (Table 1), and after the addition of the simulated salivary fluid (SSF), the pH was not re-adjusted to 7 due to the short duration at the oral phase. Therefore, GDL gels had a slightly acidic pH in the salivary phase with more phenolic acid molecules being in the protonated form, which makes them less water-soluble [19].

Overall, the release profiles of the different phenolic acids were significantly different (*p* < 0.05). The release of PCA was faster than the CMA from the end of the oral processing. Around 90% of the PCA was initially released, slowly reducing to 80% where it remained relatively constant with a slight increase during the intestinal phase (*p* > 0.05). The modulation of CMA release was more gradual than PCA during the oral and gastric phase, with a dramatic increase at the intestinal phase (*p* < 0.05). The release percentage of CMA reached almost 100% and remained stable throughout intestinal processing.

In a study where a cheddar-like cheese with incorporated tea polyphenols was developed, it was found that even though the levels of total polyphenols were low in stomach conditions, they gradually increased during the first 40 min of the intestinal processing [20]. Unfortunately, the authors did not characterize the tea extract and they only measured the total polyphenols with a photometric method [20]. Several studies have demonstrated significant losses in endogenous phenolic acids during in vitro digestion of pomegranate products [68], whole grapes [69], and broccoli [70]. Therefore, our results signify that pressed soy protein gels are promising food systems for the delivery of phenolic acids. More research is needed, however, to determine the exact features responsible for the protective effect of soy protein gels on phenolic acids observed here.

## 4. Conclusions

The two types of gels were similar in composition, but they had significant differences both in texture and microstructure. MgSO_4_-induced gels were more porous with larger aggregates, but they had significantly lower firmness than the GDL-induced gels, which might be the reason for their higher protein digestibility. The release of the bioactives on a percentage basis was similar for both gel matrices, but GDL-induced gels delivered larger masses of bioactives in the intestinal phase because they had the capacity to retain more of the phenolics. Our results suggested that the coagulation mechanism affected both the proteolysis of the soy protein gels and the bioaccessibility of added phenolic acids.

## Figures and Tables

**Table 1:** Physical characteristics of two types of pressed soy protein gels induced by glucono δ-lactone (GDL) and MgSO_4_ with added phenolic acids: protocatechuic acid (PCA) and coumaric acid (CMA). Values were represented as means ± standard deviations (*n* = 3). ^a–d^ Different letters within the same row of six values indicate significant differences (*p* ≤ 0.05).

	GDL-INDUCED GELS			MgSO_4_-INDUCED GELS		
Properties	Control	PCA	CMA	Control	PCA	CMA
Moisture (%)	83.6 ± 0.43 ^ab^	84.2 ± 0.80 ^abc^	83.4 ± 0.34 ^a^	86.2 ± 0.74 ^bd^	85.2 ± 0.38 ^cd^	85.7 ± 0.11 ^cd^
Yield (%)	31.9 ± 2.17 ^a^	33.1 ± 5.15 ^a^	34.1 ± 3.51 ^a^	38.7 ± 5.18 ^a^	37.1 ± 3.12 ^a^	37.9 ± 3.30 ^a^
Protein in curd (%)	15.1 ± 0.83 ^a^	14.7 ± 0.48 ^ab^	15.4 ± 0.38 ^a^	13.2 ± 1.05 ^bc^	13.2 ± 0.40 ^bc^	12.3 ± 0.54 ^c^
Soluble protein in whey (%)	0.13 ± 0.02 ^a^	0.13 ± 0.02 ^a^	0.12 ± 0.00 ^a^	0.36 ± 0.03 ^b^	0.35 ± 0.05 ^b^	0.30 ± 0.05 ^b^
Whey (%)	67.0 ± 2.97 ^a^	65.9 ± 4.23 ^a^	64.5 ± 0.83 ^a^	60.9 ± 4.66 ^a^	63.9 ± 2.57 ^a^	63.3 ± 2.93 ^a^
pH of whey (-)	4.7 ± 0.08 ^a^	4.7 ± 0.11 ^a^	4.8 ± 0.06 ^a^	6.4 ± 0.03 ^b^	6.1 ± 0.04 ^c^	6.1 ± 0.07 ^c^
Zeta potential of whey (mV)	−3.4 ± 0.21 ^a^	−2.0 ± 0.05 ^b^	−2.7 ± 0.04 ^cd^	−2.5 ± 0.07 ^d^	−2.6 ± 0.03 ^d^	−3.1 ± 0.11 ^ac^
Retention of PA in curd (%)	n/a	40.2 ± 6.62 ^a^	58.8 ± 3.59 ^b^	n/a	35.0 ± 6.72 ^a^	43.8 ± 6.17 ^a^

## Supplementary Materials

The following are available online at https://www.mdpi.com/2304-8158/10/1/154/s1, Figure S1: Mass balance diagram of gelation procedure of GDL-CMA and MgSO_4_-CMA pressed gels. Protein and bounded phenolic acid concentration after pressing depends on coagulant and used. Table S1: Free amino acid concentration (μmol/g of digesta at the intestinal phase) of in vitro blank digestions (without substrate) and pressed control samples induced by GDL and MgSO_4_.
