# Supplementary material for: Soy Protein Pressed Gels: Gelation Mechanism Affects the In Vitro Proteolysis and Bioaccessibility of Added Phenolic Acids
Source: Foods. 2021 Jan 13;10(1):154. doi: 10.3390/foods10010154 (PMC7828434; doi:10.3390/foods10010154)
Supplement: Supplementary file 1 [file foods-10-00154-s001.zip › Table S1.pdf]

foods

MDPI

Table S1. Free amino acid concentration (μmol/ g of digesta at the intestinal phase) of in vitro blank digestions (without substrate) and pressed control samples induced by GDL and $MgSO_4$. Values were represented as means ± standard deviations (n=3-4). Different letters within the same row indicate significant differences ($p \leq 0.05$). Values were represented as means ± standard deviations (n=3-4). Different letters within the same row indicate significant differences ($p \leq 0.05$).

	Concentration (μmol/ g of Digesta)		
**AA**	**Blank**	**$MgSO_4$ gels**	**GDL gels**
Aspartic acid	1.1 ± 0.2 [a]	0.8 ± 0.1 [a]	0.5 ± 0.1 [b]
Glutamic acid	2.6 ± 0.6 [a]	2.5 ± 0.4 [a]	1.3 ± 0.2 [b]
Serine	2.9 ± 1.0 [a]	2.9 ± 0.3 [a]	1.6 ± 0.3 [a]
Histidine	0.8 ± 0.1 [b]	1.4 ± 0.2 [a]	0.7 ± 0.3 [b]
Glycine	4.1 ± 1.9 [a]	3.0 ± 0.3 [a]	1.8 ± 0.6 [a]
Threonine	1.9 ± 0.5 [ab]	2.1 ± 0.2 [a]	1.1 ± 0.2 [b]
Arginine	3.4 ± 1.0 [b]	6.5 ± 0.7 [a]	4.0 ± 1.2 [b]
Alanine	3.3 ± 1.2 [a]	3.6 ± 0.4 [a]	2.2 ± 0.3 [a]
Tyrosine	2.4 ± 0.6 [a]	3.3 ± 0.5 [a]	2.3 ± 0.3 [a]
Valine	1.9 ± 0.2 [b]	2.6 ± 0.3 [a]	1.3 ± 0.2 [b]
Methionine	0.6 ± 0.0 [b]	0.9 ± 0.1 [a]	0.6 ± 0.1 [b]
Phenylalanine	1.4 ± 0.3 [a]	5.0 ± 0.7 [c]	3.2 ± 0.6 [b]
Isoleucine	1.4 ± 0.3 [b]	2.9 ± 0.3 [a]	1.7 ± 0.2 [b]
Leucine	3.4 ± 1.5 [b]	8.1 ± 1.0 [a]	4.8 ± 1.2 [b]
Lysine	8.0 ± 7.2 [a]	6.5 ± 0.5 [a]	3.9 ± 1.3 [a]

© 2020 by the authors. Submitted for possible open access publication under the terms and conditions of the Creative Commons Attribution (CC BY) license (http://creativecommons.org/licenses/by/4.0/).
